# Cutaneous vascular & sudomotor responses to heat-stress in smokers & non-smokers

**DOI:** 10.1186/2046-7648-4-S1-A98

**Published:** 2015-09-14

**Authors:** Nicole E Moyen, Hannah A Anderson, Jenna M Burchfield, Matthew A Tucker, Melina A Gonzalez, Forrest B Robinson, Matthew S Ganio

**Affiliations:** 1 Human Performance Laboratory, Department of Health, Human Performance, and Recreation, University of Arkansas, Fayetteville, AR, USA

## Introduction

As approximately one billion people worldwide are chronic smokers [1] it is important to determine smokers' thermoregulatory responses to heat-stress. Although local maximal vasodilation may be attenuated in smokers [2], skin blood flow responses during whole-body heat stress are unknown. Moreover, it is unknown if sweat rate is altered in smokers; theoretically the binding of nicotine to nicotinic acetylcholine receptors [2] may initiate an earlier onset of sweating during whole-body heat stress compared to non-smokers [3]. The purpose of this study was to compare cutaneous vascular and sudomotor responses to whole-body passive heat-stress between smokers and non-smokers.

## Methods

Nine male chronic smokers [SMK; 10 (6) cigarettes/day for 11.8 (9.5) y; 26 (8) y; 177.7 (6.6) cm; 80.6 ± 21.1 kg] and 13 male non-smokers [N-SMK; 28 (9) y; 177.6 (6.8) cm; 77.2 (8.2) kg] were matched for age, height, body mass, and exercise habits (all p > 0.05). Subjects were passively heated via water-perfused suits until gastrointestinal temperature (T_gi_) increased 1.5 °C. Local sweat rate (LSR) via ventilated capsule and cutaneous vasomotor activity (CVC) via Laser Doppler on the forearm were continuously recorded; blood pressure, heart rate, sweat gland activation (SGA), sweat gland output (SGO), T_gi_, and mean-weighted skin temperature (T_sk_) were taken at baseline and each 0.5 °C T_gi _increase. LSR and CVC onsets and sensitivities were calculated with mean body temperature (T_b_) = 0.9*T_gi _+ 0.1*T_sk _[4]_._

## Results

No differences existed between SMK and N-SMK for T_gi_, T_sk_, T_b_, heart rate, mean arterial pressure, LSR, CVC, and SGA with each 0.5 °C T_gi _increase (all p > 0.05). Overall, SGO tended to be lower in SMK than N-SMK [SMK = 5.94 (3.49) vs. N-SMK = 8.94 (3.99) µg·gland^-1^·min^-1^; p = 0.08].

## Discussion

Smokers' CVC and LSR onsets occurred at an earlier T_b _than non-smokers, possibly because heat stress enhances nicotine kinetics (*i.e*. binding of nicotine to nicotinic acetylcholine receptors; [2,3]). The lower LSR at plateau during whole-body heating might indicate a thermoregulatory impairment in young smokers, and is likely a result of decreased sweat gland output and not activation.

## Conclusion

Compared to non-smokers, smokers had an earlier onset but similar sensitivity (i.e. increase in response per increase in T_b_) for sweating/cutaneous vasodilation. These data suggest that overall, most young chronic smokers' thermoregulatory responses to whole-body passive heat stress are not impaired.

**Table 1:** Mean (SD) CVC and LSR parameters on the forearm for SMK and N-SMK during passive heat stress

Measurement		Smokers	Non-smokers
**CVC**	CVC onset (ΔT_b _from baseline, °C)	0.31 (0.12)	0.61 (0.21)*

	CVC plateau (% of max)	68.4 (27.4)	68.4 (21.6)

	CVC sensitivity (Δ%max per °C ΔT_b_)	82.5 (46.2)	58.9 (23.3)

**LSR**	LSR onset (ΔT_b _from baseline, °C)	0.35 (0.14)	0.52 (0.19)*

	LSR plateau (mg·cm^-2^·min^-1^)	0.79 (0.26)	1.00 (0.13)*

	LSR sensitivity (Δmg·cm^-2^·min^-1 ^per °C ΔT_b_)	0.60 (0.40)	0.63 (0.21)

*Significant difference between groups (p < 0.05).
